# Cytokine Expression Profile of Dengue Patients at Different Phases of Illness

**DOI:** 10.1371/journal.pone.0052215

**Published:** 2012-12-20

**Authors:** Anusyah Rathakrishnan, Seok Mui Wang, Yongli Hu, Asif M. Khan, Sasheela Ponnampalavanar, Lucy Chai See Lum, Rishya Manikam, Shamala Devi Sekaran

**Affiliations:** 1 Department of Medical Microbiology, Faculty of Medicine, University of Malaya, Kuala Lumpur, Malaysia; 2 Institute of Medical Molecular Biotechnology, Faculty of Medicine, Universiti Teknologi MARA, Selangor, Malaysia; 3 Perdana University Graduate School of Medicine, Serdang, Selangor, Malaysia; 4 Department of Pharmacology and Molecular Sciences, The Johns Hopkins University School of Medicine, Baltimore, Maryland, United States of America; 5 Department of Infectious Diseases, University Malaya Medical Centre, Kuala Lumpur, Malaysia; 6 Department of Paediatrics, Faculty of Medicine, University of Malaya, Kuala Lumpur, Malaysia; 7 Department of Trauma and Emergency Medicine, University Malaya Medical Centre, Kuala Lumpur, Malaysia; University of Sao Paulo, Brazil

## Abstract

**Background:**

Dengue is an important medical problem, with symptoms ranging from mild dengue fever to severe forms of the disease, where vascular leakage leads to hypovolemic shock. Cytokines have been implicated to play a role in the progression of severe dengue disease; however, their profile in dengue patients and the synergy that leads to continued plasma leakage is not clearly understood. Herein, we investigated the cytokine kinetics and profiles of dengue patients at different phases of illness to further understand the role of cytokines in dengue disease.

**Methods and Findings:**

Circulating levels of 29 different types of cytokines were assessed by bead-based ELISA method in dengue patients at the 3 different phases of illness. The association between significant changes in the levels of cytokines and clinical parameters were analyzed. At the febrile phase, IP-10 was significant in dengue patients with and without warning signs. However, MIP-1β was found to be significant in only patients with warning signs at this phase. IP-10 was also significant in both with and without warning signs patients during defervescence. At this phase, MIP-1β and G-CSF were significant in patients without warning signs, whereas MCP-1 was noted to be elevated significantly in patients with warning signs. Significant correlations between the levels of VEGF, RANTES, IL-7, IL-12, PDGF and IL-5 with platelets; VEGF with lymphocytes and neutrophils; G-CSF and IP-10 with atypical lymphocytes and various other cytokines with the liver enzymes were observed in this study.

**Conclusions:**

The cytokine profile patterns discovered between the different phases of illness indicate an essential role in dengue pathogenesis and with further studies may serve as predictive markers for progression to dengue with warning signs.

## Introduction

In certain infectious diseases, shock may occur due to excessive plasma leakage and this leakage is often postulated to be caused by endothelial sieves created by inappropriate cytokine responses in the host. Dengue, traditionally classified as Dengue Fever (DF), Dengue Haemorrhagic Fever (DHF) and Dengue Shock Syndrome (DSS) is one such disease where a key feature of DHF is vascular leakage which then leads to hypovolemic shock (DSS), inevitably increasing the chances of fatality. Recently, WHO has suggested a new classification for this disease, which includes dengue with or without warning signs and severe dengue [1].

Dengue, although with a low mortality rate, is one of the highest morbidity rated arthropod diseases. It is endemic in more than 120 countries around the world, with 55% of the world’s population at risk of being infected [1]. Despite being around for centuries, there have not been any effective vaccines, therapeutics or anti-viral drugs for this disease. The lack of such “cure” can be attributed to firstly, to an incomplete understanding of dengue immunopathogenesis, secondly, a lack of a suitable animal model and finally the inherent dangers of live vaccines [2].

Some of the postulated hypotheses on dengue immunopathogenesis include (i) the antibody enhancement theory [3], [4], (ii) cross-reactive memory T cells activation [5] and (iii) the original antigenic sin [6], where all in a way cause either an over production or a skewed profile of cytokine release, hence the term cytokine storm/cytokine tsunami. This cytokine storm has a direct effect on the vascular endothelial cells by increasing capillary permeability and causing leakage [7]. Cytokines also exhibit synergism, where for example, tumor necrosis factor-alpha (TNF-α), interferon-γ (IFN-γ) and interleukin-1 (IL-1) together can increase the capillary permeability compared to when the cytokine is acting alone [8]. There is also the inapparent ability of the endothelium to repair itself which could be a result of some aspect of endothelial dysfunction, though this has not been shown.

The study of permeability and leakage in dengue is often applied with the use of human umbilical vein endothelial cell (HUVEC) line where in one such study, Anderson *et al*. observed that these cells are activated when exposed to culture fluids from dengue virus (DENV)-infected peripheral blood mononuclear cells (PBMCs) [9]. In another study, the vascular permeability of HUVECs was found to be increased when exposed to either recombinant human monocyte chemo-attractive protein-1 (rhMCP-1) or to the culture supernatant of DENV2-infected human monocytes [10]. Moreover, certain cell lines, such as DENV-infected primary human monocytes and epithelial cell lines have shown increased production of cytokines [11]. DENV infections of HepG2 and primary dendritic cells (DCs) have also shown the ability to induce the production of cytokines such as IL-8, RANTES, macrophage inhibitory protein-1-alpha (MIP-1α) and MIP-1β [12].

The cytokine storm hypothesis has also been studied by analyzing sera of DHF/DSS patients in Vietnam, India and Cuba, which indeed showed the presence of elevated levels of IFN-γ, TNF-α and IL-10 [13], [14], [15]. A recent study on dengue infected Venezuelan patients had documented significant increased levels of MCP-2, IP-10 and TRAIL in patients’ serum during the febrile period [16].

Despite extensive research on the role of cytokines in the progression of severe dengue [17], [18], [19], [20], the cytokine profiles, especially at the defervescence stage, and the synergy between them that leads to continued plasma leakage is not clearly understood. Thus, in this study, not only did we attempt cytokine profiling of dengue patients, but also set to establish patient and cytokine kinetics throughout the phases of illness. We opted to gain preliminary insights on differences in the levels of cytokines in primary and secondary dengue infections. With that, we endeavoured to analyse the relationship between the cytokines and the clinical parameters of dengue patients.

## Materials and Methods

### Ethics Statement and Study Population

Five millilitres of blood were obtained from 44 DENV infected patients at the University Malaya Medical Centre (UMMC), Kuala Lumpur, Malaysia from January 2005 to June 2009 with written informed consent. Blood was drawn at three time point of illness- febrile, defervescence and convalescence for each patient. The febrile phase usually lasts for 2–7 days and often has indistinguishable clinical symptoms, whereas defervescence is the critical stage where patient may develop severe signs and the convalescence stage is when the patient starts to recover. The 2009 WHO dengue classification scheme and case definition [1] was used to diagnose patients. Data on demographic characteristics (i.e. age, gender and race), clinical features (i.e. day of fever, body temperature, bleeding manifestation, leakage, abdominal pain and hypotension) and routine haematological and biochemical laboratory test findings (i.e. full blood count, liver function tests) were also collected. Healthy donors’ blood samples that are of age, gender and race matched with patients, were obtained from Blood Bank, UMMC as controls. All data analysed were anonymized and ethical clearance for this work was approved by the Scientific and Ethical Committee of UMMC (Ethics Committee/IRB Reference No: 321.4).

### Sera Isolation and Dengue Confirmatory Tests

Blood serum was collected by centrifugation of blood containing tubes at 1500 rpm for 10 minutes and the serum was stored at −80°C until further use. All patients were further confirmed to have dengue by detection of (i) DENV via virus isolation; (ii) DENV RNA via real-time SYBR-Green based RT-PCR assay [21]; (iii) DENV antigen via NS1 assay (Pan-E dengue early ELISA kit; Panbio, Queensland, Australia); (iv) DENV-specific antibodies via in-house capture IgM Enzyme-Linked Immunosorbent Assay (ELISA) [22] and haemagglutination inhibition (HI) test [23]. The HI assay was also used to define primary and secondary DENV infection based on the total antibody in paired sera.

### Identification and Quantification of Cytokines

Twenty-nine different cytokines, namely IL-1β, IL-1ra, IL-2, IL-4, IL-5, Il-6, IL-7, IL-8, IL-9, IL-10, IL-12, IL-13, IL-15, IL-17, IL-18, IP-10, ICAM-1, IFN-γ, MCP-1, MIP-1α, MIP-1β, Eotaxin, basic-fibroblast growth factor (FGF-Basic), granulocyte colony-stimulating factor (G-CSF), granulocyte macrophage colony-stimulating factor (GM-CSF), platelet-derived growth factor (PDGF), vascular endothelial growth factor (VEGF), RANTES, TNF-α were evaluated. The cytokine levels in the serum of all patients and controls, at all 3 different phases of illness (febrile, defervescence, and convalescence) were analyzed using the Bio-Plex human cytokine 27-plex panel, 8-plex panel and 2-plex panel kits (Bio-Plex Human Cytokine Assay; Bio-Rad Inc., Hercules, CA, USA). Briefly, patients’ serum samples were mixed with beads coated with antibodies (Abs) to various cytokines, having unique fluorescent intensity. Subsequently, the mixtures were incubated with biotinylated anti-cytokine Abs. Finally, PE-conjugated streptavidin was added, and the fluorescent signals were detected using the multiplex array reader Bio-Plex 200 System (Bio-Rad Laboratories). Raw data was initially measured as the relative fluorescence intensity and then converted to cytokines concentration based on the standard curve generated from the reference concentrations supplied in the kit (Bio-Rad Laboratories).

### Statistical Analysis

The Kruskal-Wallis one way analysis of variance (ANOVA), followed by Dunn’s multiple comparison test were used to evaluate differences between raw cytokine levels in the different groups of dengue patients compared to control groups. The Mann-Whitney U Test was applied to assess differences in the cytokine levels of primary and secondary infections. To establish the correlation between cytokine levels and clinical parameters/findings, the correlation matrix was applied. Results are given as correlation coefficient, r (ranges from −1 to +1). Two-tailed P value of less than 0.05 was considered to be significant for all test performed. All three statistical analyses performed were done using GraphPad Prism 5 for Windows, Version 5.01 (San Diego, California, USA).

## Results

### Characteristics of Study Population

Forty-four adult patients with laboratory confirmed dengue virus infection were investigated for their cytokine profiles. These patients were classified by the WHO 2009 guideline into 11 with “Dengue without Warning Signs (DwoWS)”, 29 with “Dengue with Warning Signs (DwWS) and 4 with “Severe Dengue (SD)”. The 24 males and 20 females study cohort consisted of 24 Malays, 4 Chinese, 13 Indians and 3 of other ethnicities. The demographics and clinical parameters (age, duration of illness, temperature, platelet count, and hematocrit) as well as clinical symptoms (bleeding manifestation, plasma leakage, abdominal pain and hypotension) are described in Table 1 and Table 2.

**Table 1:** Demographics of study cohort.

Demographics	Number/Range (Mean)		
	DwoWS	DwWS	SD
**Number of patients**	11	29	4
**Gender**	F-6; M-5	F-11; M-18	F-3; M-1
**Age (years)**	14–57 (29)	14–67 (30)	15–60 (39)
**Duration of illness (days)**	2–15 (6)	2–14 (6)	4–13 (7)
**Body temperature (°C)**	36.0–40.3 (37.5)	35.8–40.2 (37.8)	35.8–39.7 (37.8)

DwoWS: Dengue without Warning Signs; DwWS: Dengue with Warning Signs; SD: Severe Dengue; F: Female; M: Male.

**Table 2:** Clinical symptoms experienced by study population.

Clinical Symptoms	Number of Patients		
	DwoWS	DwWS	SD
**Abdominal pain**	0	16	2
**Hypotension**	2	6	2
**Bleeding manifestation** *	7	18	4
**Plasma leakage**	0	27	4
**Co-morbidities** #	0	3	1

DwoWS: Dengue without Warning Signs; DwWS: Dengue with Warning Signs; SD: Severe Dengue;

* bleeding manifestation in DwoWS does not include mucosal bleeding;

# Co-morbidities observed include diabetes mellitus, hypertension, hypercholesterolemia and asthma.

The liver function tests (Table 3) included measurement of total bilirubin (TB), total albumin (TA), aspartate transaminase (AST), alanine transaminase (ALT), alkaline phosphatase (ALP) and gamma glutamyl transferase (GGT). Whereas, the white blood cell (WBC) profile (Table 4) of this study population includes WBC, neutrophils, lymphocytes, monocytes and atypical lymphocytes.

**Table 3:** Liver enzyme profile of study population.

Liver enzymes	Difference vs normal levels (mean levels)									
	Normal	DwoWS			DwWS			SD		
	Level (Range)	Feb	Def	Conv	Feb	Def	Conv	Feb	Def	Conv
**Total Bilirubin (µmol/L)**	3–17	–	–	–	–	–	–	–	↑ (25.2)	↑ (50.5)
**Total Albumin (g/L)**	35–50	–	–	–	–	–	–	↓ (32)	↓ (27.7)	↓ (29.5)
**Aspartate Transaminase (AST) (IU/L)**	15–37	↑ (117.4)	↑ (179.2)	↑ (71.8)	↑ (121.6)	↑ (144.5)	↑ (125.6)	↑ (115.5)	↑ (778.8)	↑ (530.5)
**Alanine Transaminase (ALT) (IU/L)**	30–65	↑ (115.0)	↑ (155.5)	↑ (107.5)	↑ (101.2)	↑ (135.4)	↑ (149.8)	↑ (80)	↑ (207.7)	↑ (268.5)
**Alkaline phosphatase (ALP) (IU/L)**	50 to 136	–	–	–	–	–	–	–	↑ (204.5)	↑ (201.5)
**Gamma glutamyl transferase (Gamma-GT) (IU/L)**	15 to 85	–	↑ (126.1)	–	–	↑ (127.1)	–	–	↑ (234.8)	↑ (194)

DwoWS: Dengue without Warning Signs; DwWS: Dengue with Warning Signs; SD: Severe Dengue; Feb: Febrile; Def: Defervescence; Conv: Convalescence; ↓: decrease in mean levels; ↑: increase in mean levels; –indicates no mean differences compared to the normal levels.

**Table 4:** Blood and white blood cells profile of study population.

Blood and White Blood Cells	Difference vs normal levels (mean levels)									
	Normal	DwoWS			DwWS			SD		
	Level(Range)	Feb	Def	Conv	Feb	Def	Conv	Feb	Def	Conv
**Platelet (10^6^/mL)**	150–400	–	↓ (108.3)	–	↓ (119.70)	↓ (81.68)	–	↓ (131.5)	↓ (57.17)	–
**HCT (%)**	36–50	–	–	–	–	–	–	–	–	↓ (32.5)
**White Blood Cells (WBC) (×10^9^/L)**	4–11	–	–	–	↓ (3.66)	–	–	–	–	–
**Neutrophils (%)**	40–75	–	–	↓ (36.25)	–	–	–	–	–	↓ (38.00)
**Lymphocytes (%)**	20–45	–	–	↑ (47)	–	–	–	–	–	↑ (54.00)
**Monocytes (%)**	2 to 10	↑ (10.17)	–	↑ (10.25)	–	↑ (10.43)	↑ (12.00)	–	–	–
**Atypical lymphocytes (%)**	0 to 1	–	↑ (6.83)	–	↑ (6.75)	↑ (12.00)	↑ (10.50)	–	↑ (6.25)	–

DwoWS: Dengue without Warning Signs; DwWS: Dengue with Warning Signs; SD: Severe Dengue; Feb: Febrile; Def: Defervescence; Conv: Convalescence; ↓: decrease in mean levels; ↑: increase in mean levels; –indicates no mean differences compared to the normal level.

From the lab diagnostic tests performed (Table 5), DENV was successfully isolated from 17 patients and DENV RNA was detected in 31 patients. All the 4 DENV serotypes were detected in our study cohort, with the highest percentage to be DENV-1 (43.3%) and the rest ranging from 13.3%–23.3%. DENV NS1 antigen was detected 27 patients. Serologically, 41 patients were found to be IgM positive and amongst them, 37 had IgM seroconversion during the defervescence or convalescence stage. From the HI test, 16 patients had primary infections whereas the rest were experiencing either presumptive or confirmed secondary infections.

**Table 5:** Laboratory diagnostic assay results in the study cohort.

Laboratory diagnostic assays	DwoWS	DwWS	SD
Virus Isolation	2	12	3
DENV RNA detection	5	22	4
DENV NS1 Antigen detection	5	19	3
DENV IgM detection	10	28	3
Seroconversion	9	27	1
Haemagglutination Inhibition	11	29	4
Primary	4	11	1
Secondary	7	18	3

DwoWS: Dengue without Warning Signs; DwWS: Dengue with Warning Signs; SD: Severe Dengue.

### Levels of Cytokines at Different Phases of Illness

The levels of 29 different types of cytokines in dengue patients were determined at the febrile, defervescence and convalescent phases. In this analysis, the 4 severe dengue patients were excluded due to the small sample size. We then categorized the cytokines into 3 groups: (i) inflammatory cytokines; (ii) chemokines; (iii) adhesion molecules and growth factors. The cytokines which demonstrated significant differences between the different groups of patients and controls are as summarized in Table 6.

**Table 6:** Significant cytokines at different phases of illness when compared to the controls.

Cytokines	Controls (pg/ml)	Acute (pg/ml)	Defervescence (pg/ml)	Convalescence
**IP-10**	1114.69±250.45	DwoWS ↑↑ (43516.26±12188.23)	DwoWS ↑↑ (35574.12±6509.15)	–
		DwWS ↑↑ (58880.34±7867.14)	DwWS ↑↑ (26983.01±3042.01)	
**MCP-1**	84.11±12.80	DwWS ↑↑ (438.87±91.41)	–	–
**MIP-1β**	102.49±12.53	DwWS ↑↑ (662.81±380.06)	DwoWS ↑↑ (618.36±163.05)	–
**G-CSF**	65.31±13.16	–	DwoWS ↓↓ (23.56±2.05)	–

DwoWS: Dengue without Warning Signs; DwWS: Dengue with Warning Signs; ↑↑: significant increase compared to controls (p<0.05); ↓↓: significant decrease compared to controls (p<0.05); – : no significant changes observed between group.

#### i. Inflammatory cytokines (Figure 1A)

Fifteen different inflammatory cytokines were analyzed where the majority of them were pro-inflammatory while only 4 were anti-inflammatory. Of these, various trends were observed, notably, among the pro-inflammatory cytokines, with only the mean cytokine levels of IL-18 were elevated for both groups of patients at all three phases of illness. The levels of pro-inflammatory cytokines, IL-5 and IL-12 as well as anti-inflammatory cytokine IL-4 were lower throughout the illness in both groups when compared to the healthy controls.

**Figure 1:**
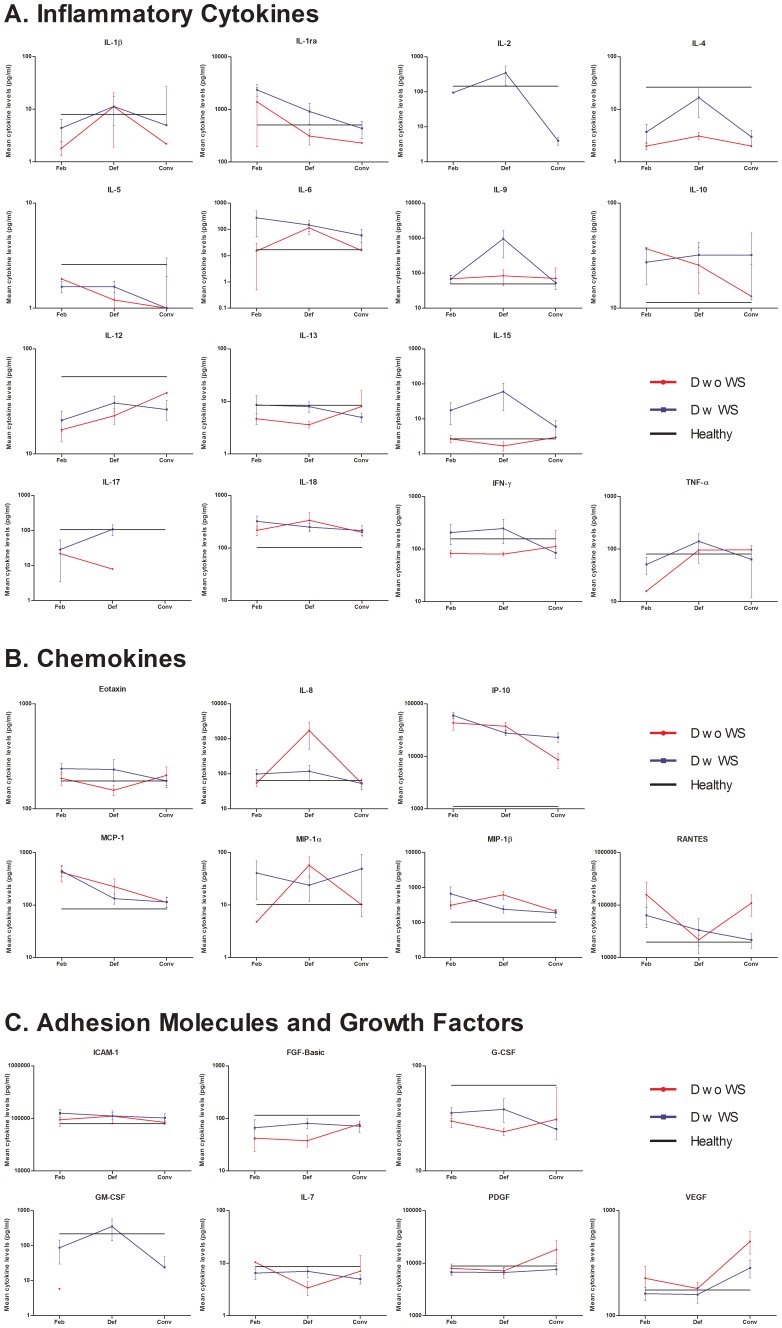
Cytokine trends over the three different phases of illness. The mean levels of (A) inflammatory cytokines, (B) chemokines and (C) adhesion molecules and growth factors in dengue patients without warning signs (DwoWS) (red symbol), dengue patients with warning signs (DwWS) (blue symbol) and healthy controls (black line) at different phase of illness (Feb = Febrile; Def = Defervescence; Conv = Convalescence). Error bars indicate standard error mean (SEM).

The IFN-γ levels were generally lower in DwoWS patients and were higher in DwWS patients with decreasing trend as patients recovered. The cytokines IL-1β and TNF-α displayed a similar trend where lower levels were detected during febrile phase, which then peaked during defervescence. The levels of IL-6 were generally higher in DwWS patients, but during defervescence, DwoWS patients had high levels similar to the warning signs group. Both groups displayed higher levels of IL-9 than controls, but the patients with warning signs had peak levels during defervescence. Anti-inflammatory cytokines, IL-10 and IL-13 showed mixed patterns, whereas IL-1ra in both groups displayed similar a trend throughout the disease, however with the warning signs group having higher levels than the DwoWS patients. Two cytokines (IL-2 and IL-17) had insufficient patient response in both groups. Despite the various trends observed, none of the inflammatory cytokines were significantly different between the groups studied at any time point.

#### ii. Chemokines (Figure 1B)

**Figure 2:**
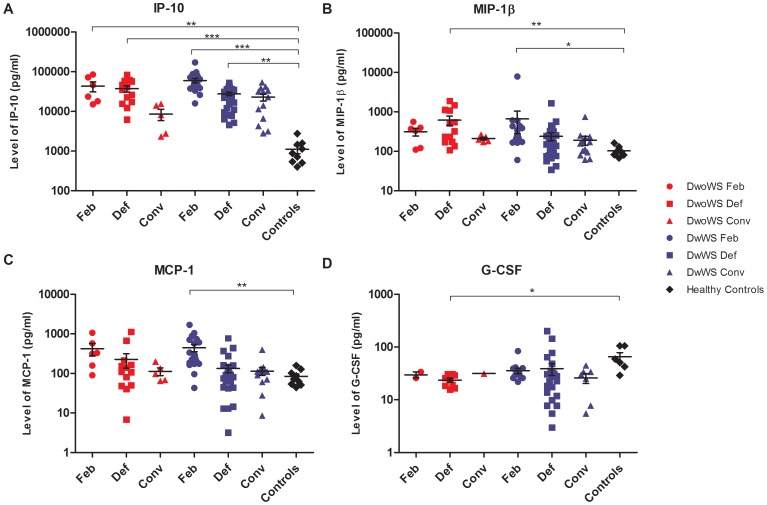
Distribution of raw cytokine response values in dengue without warning signs (DwoWS) and dengue with warning signs (DwWS) compared with healthy controls. Levels of (A) IP-10, (B) MIP-1β, (C) MCP-1 and (D) G-CSF in DwoWS (red symbol), DwWS (blue symbol) patients at different phase of illness compared with control subjects (black symbol). The SEM are indicated by the error bars in the scatter plot. Statistical significance based on the Kruskal-Wallis, Dunn’s multiple comparison test where *-*P*<0.05; **-*P*<0.01, and ***-*P*<0.001.

More than half of the chemokines analysed, namely IP-10, MCP-1, MIP-1β, and RANTES, had elevated mean cytokine levels in both patient with and without warning signs across the three time points. Notably, IP-10 levels in both groups were significantly different from healthy donors at febrile (DwoWS: P<0.01; DwWS: P<0.001) and defervescence (DwoWS: P<0.001; DwWS: P<0.01) phases (Figure 2A). In the case of MIP-1β, a significant difference was noted in the DwoWS during defervescence (P<0.01) and in the DwWS patients in the febrile phase (P<0.05) of disease compared to the controls (Figure 2B). Further, the MCP-1 was significantly higher (P<0.01) in the warning signs patients during the febrile stage when compared to the controls (Figure 2C). Eotaxin was generally lower in DwoWS patients and higher in DwWS patients and IL-8 was higher in patients without warning signs compared to the DwWS.

#### iii. Adhesion molecules and growth factors (Figure 1C)

The only adhesion molecule studied, ICAM-1, had mean levels that were slightly elevated in both groups of patients. In contrast, the mean levels of growth factors, FGF-Basic and G-CSF, were decreased in both groups relative to normal groups at all the three phases of illness, with a significant decrease for G-CSF in the patients’ without warning signs (P<0.05) (Figure 2D) during defervescence. Other growth factors, IL-7, PDGF and VEGF were generally lower in patients with warning signs, relative to both patients without warning signs and the normal group. Growth factor GM-CSF lacked data for in group without warning signs.

### Levels of Cytokines in Primary Versus Secondary Infections

**Figure 3:**
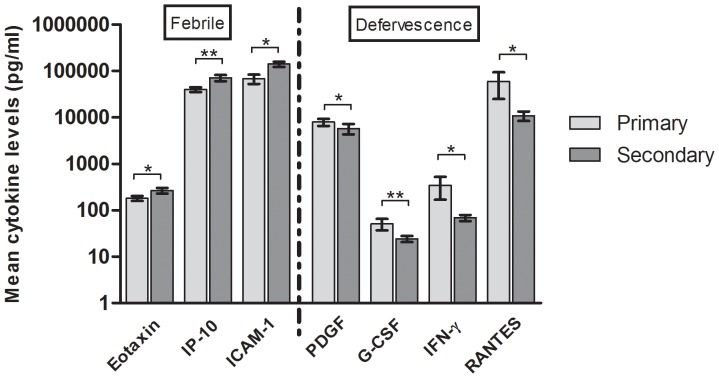
Comparison of mean cytokine levels in primary and secondary dengue infections. Primary and secondary infection status was determined by haemagglutination inhibition assay. Mean with SEM of seven cytokines (three at febrile phase of illness and 4 at defervescence). Statistical significance based on the Mann-Whitney U test where *-*P*<0.05 and **-*P*<0.01.

The differences in cytokines levels were also assessed between the 16 primary infected patients and the 28 patients who were experiencing secondary infections. Of the 29 cytokines analyzed, most displayed relatively similar levels in both infection statuses. However, at the febrile phase, we found that 3 cytokines differed significantly where eotaxin, IP-10 and ICAM-1 were significantly higher in patient with secondary infections (Figure 3). At the defervescence phase, on the other hand, pro-inflammatory cytokine IFN-γ, chemokine RANTES and growth factors, PDGF as well as G-CSF, were found to be significantly higher in primarily infected patients than those suffering from secondary infections (Figure 3).

### Relationship between Cytokines and Clinical Parameters

The possible associations between the levels of cytokines with clinical presentation of our study cohort are described in Table 7. Generally, in patients without warning signs, we found that the decreased levels of platelet were associated with the decreasing levels of VEGF and RANTES during defervescence. At this phase, the augmented liver enzymes were associated with several cytokines where AST was associated with increased levels of IL-1ra and IL-10; ALT with IP-10 and Gamma-GT with IL-4, IL-12 and IL-9. In the convalescence phase, the increased levels of AST were conversely related to the decreasing levels of MCP-1. Whereas, the increased levels of VEGF at this time point, was associated with the decreased levels of neutrophils and higher state of lymphocytes.

**Table 7:** Association of cytokines with clinical parameters in the study cohort.

Clinical Manifestations	Coefficient (r)	Clinical Manifestations	Coefficient (r)
**DwoWS (Defervescence)**		**DwWS (Febrile)**	
**Platelet:**		**Platelet:**	
VEGF	0.862**	IL-7	0.946***
RANTES	0.740*	IL-12	0.607*
**AST:**		PDGF	0.524*
IL-1ra	0.764*	**AST:**	
IL-10	0.756*	ICAM-1	0.803*
**ALT:**		FGF-Basic	−0.898*
IP-10	0.574*	IL-13	−0.805**
**Gamma-GT:**		IL-4	−0.690*
IL-4	0.850**	IL-12	−0.636*
IL-12	0.752*	VEGF	−0.555*
IL-9	0.713*	**DwWS (Defervescence)**	
**DwoWS (Convalescence)**		**Platelet:**	
** AST:**		IL-7	0.892***
MCP-1	−0.980*	PDGF	0.867***
**Neutrophils:**		IL-5	0.662***
VEGF	−0.960*	RANTES	0.607*
**Lymphocytes:**		VEGF	0.574**
VEGF	0.994**	IL-12	0.547*
		**AST:**	
		PDGF	−0.416*
		**Atypical lymphocytes:**	
		G-CSF	−0.658*
		**DwWS (Convalescence)**	
		**AST:**	
		IFN-γ	0.964*
		IL-10	0.950*
		**Monocytes:**	
		IL-7	0.994**
		**Atypical lymphocytes:**	
		IP-10	0.975*

DwoWS: Dengue without Warning Signs; DwWS: Dengue with Warning Signs; r: Correlation Coefficient; AST: aspartate transaminase; ALT: alanine transaminase; Gamma-GT: Gamma glutamyl transferase. r = 1 indicates perfect correlation; r = 0 indicates X and Y do not vary together at all; r = −1 indicates perfect negative correlation. P values are based on two-tailed test and indicates the chances of random sampling, where *indicates P<0.05; **indicates P<0.01; ***indicates P<0.001.

Significant associations between the decreased levels of IL-7, IL-12 and PDGF with the decreased levels of platelet were observed in patients with warning signs during the febrile phase, whereas in the defervescence, another 3 cytokines (IL-5, RANTES and VEGF) were also associated with platelet levels. In defervescing DwWS patients, we also observed an association between the increased levels of atypical lymphocytes and the decreased levels of G-CSF. While during convalescence, an association was noted between the atypical lymphocytes and the decreasing levels of IP-10 in these patients. The convalescing patients with warning signs also showed association between the increased levels of IL-7 and monocytes. At all 3 phases of illness, the increased levels of liver enzyme AST was associated with various cytokines where in febrile, ALT was correlated to increased levels of ICAM-1 and decreased levels of FGF-Basic, IL-13, IL-4, IL-12 and VEGF. During defervescence, AST was associated with decreased levels of PDGF, whereas at convalescence, it was linked with the increased levels of IL-10, and IFN-γ.

## Discussion

Generally, most hypotheses explaining dengue immunopathogenesis conclude that the overproduction and/or a skewed cytokine response during the critical phase of disease causes plasma leakage and hence, a more severe manifestation of dengue. In this study, an analysis of various cytokines and their correlation with dengue disease was performed.

Despite finding no significant difference in the levels of inflammatory cytokines among patients with and without warning signs as well as with the healthy controls, we showed trends of various cytokines at the three different phases of illness. Outstandingly, IL-15 which was higher in patients with warning signs, has been known to be involved in T cell activation and proliferation, and has been shown to be required for memory CD8^+^ T cells division. In the absence of IL-2 (as noted in our study, where many patients had undetectable levels of IL-2), the levels of IL-15 is increased [24]. This could possibly enhance proliferation of dengue memory T cells.

Interleukins-4, -5, -12 and -13 were clearly lower in dengue patients than controls throughout the illness. IL-13, an effector cytokine, synergizes with IL-2 to regulate IFN-γ production [25], and low levels of this cytokine could be attributed to the low level of IFN-γ in our cohort especially in patients without warning signs. Regulatory cytokine IL-4, previously found to be increased in DHF/DSS patients [26] has been indicated to play a role in vascular permeability and with the exclusion of severe dengue patients in our study, this may reflect the lower levels of IL-4 observed. This cytokine has also been known for immunoglobulin class and subclass switch [27], and shift from T_h_1 to T_h_2 responses in severe dengue, and this could possibly explain the lower levels in patients without warning signs as they remain in a mild state of infection.

Interferon-γ has been shown to be increased in severe dengue cases [28], [29], and this is echoed in our study cohort with lower number of severe dengue cases, where IFN-γ was only slightly higher in patients with warning signs. Another possibility of these lower levels could be attributed to the low levels of IL-12, where an *in vivo* study showed that IL-12 (p40 chain) - deficient mice had decreased IFN-γ production [30]. IL-12 and IL-18 together, augment IFN-γ production by activating T_h_1 cells [31], and in our study, despite IL-18 being higher in dengue patients, still had interferon levels that were negligible, implying that IFN-γ production by IL-12 is a co-induction with IL-18 and IL-18 induces IFN-γ only when its receptor is upregulated by IL-12 [30].

Interleukin-10 showed a decreasing trend in patients without warning signs, however remained high in DwWS patients throughout the disease in concordance with several studies that have suggested IL-10 in dengue pathogenesis [15], [32]. An important modulator in vascular leakage, platelet-activating factor (PAF) and T cell apoptosis, the over-expression of IL-10 in transgenic mice have demonstrated inhibition of TNF-α production, where in our study TNF-α remained generally at a lower level.

A study in Brazil, demonstrated a correlation between MIP-1β and NK cells, suggesting its role in dengue protective mechanism [33]. This was again shown in another study where MIP-1β was higher in mild dengue than severe dengue [29], which is in line with our findings where this chemokine was significantly higher in patients without warning signs.

IP-10, an important mediator in inflammatory response, was shown to inhibit dengue infection through competitive binding of heparan sulphate on host cell membrane [34], [35]. Initially during the febrile and defervescence stage, both groups of patients demonstrated significant high levels of IP-10, however, the levels declined steadily for patients without warning signs throughout the phases. However, it remained high in patients with warning signs, offering a possibility that it may be affecting vascular permeability as IP-10 is a potent inhibitor of angiogenesis *in vivo* [36].

An MCP-1 deficient mice model was unable to switch into subclass T_h_2 responses [37] and this chemokine has been associated with permeability changes in endothelial cells, where alterations occur to the tight junctions of vascular endothelial cells and leading to plasma leakage in dengue patients [38], [39], [40]. In this study, significantly elevated levels of MCP-1 were found at the febrile phases of patients with warning signs compared to healthy individuals suggesting this chemokine as a possible biomarker in dengue patients who are going to develop more severe clinical outcome.

The infection status (primary versus secondary infections) of an individual has also been disputed to be involved in the pathogenesis of dengue, where most of the postulated theories revolve around secondary infections. In our study, the main limitation was the small sample size, hence we could not categorize infection status by the respective dengue classification and hence only decipher the cytokine levels of primary and secondary infected dengue patients as a whole at different time point of illness. Eotaxin, IP-10 and ICAM-1 were significantly higher in secondary infected dengue patients during the febrile phase of illness. Increased levels ICAM-1 have been indicated in endothelium damage and activation [41], [42], and 75% of the secondary cases in our cohort were of patients with warning signs and/or with severe dengue. Likewise, eotaxin levels which were higher in patients with warning signs have been demonstrated to increase permeability of human coronary artery endothelial cells by downregulating tight junction proteins [43]. IP-10 levels were significantly higher in the overall dengue patients, indicating a more vigorous inflammatory response in secondary infections.

During the defervescence phase, 4 other cytokines displayed significantly lower levels in secondary dengue cases, which were IFN-γ, RANTES, PDGF and G-CSF. Interferon-γ is a critical cytokine in the innate and adaptive immunity against viral infections. Lower levels of this cytokine during a secondary infection indicate defective ability to inhibit viral replication or to be immunomodulatory. RANTES recruits lymphocytes and NK cells to sites of inflammation, and in an influenza mice model deficient in RANTES/CCL5, delayed viral clearance and excessive inflammation occurred [44]. PDGF which promotes cellular proliferation and inhibits apoptosis and is an integral component for maintaining the vascular networks [45] is lower in secondary infections offering a possible explanation as to why secondary infected patients suffered from vascular leakage. G-CSF, a WBC stimulating factor, is lower in secondary infections in line with the occurrence of leukopenia and neutropenia in such cases [46], [47].

In dengue patients without warning signs, a decrease in platelets was noted during defervescence, and this was correlated strongly with RANTES and VEGF. Both RANTES, a chemokine stored in α-granules of platelets, secreted upon platelet activation [48], and VEGF, a growth factor released by platelets, would be expected to decrease upon thrombocytopenia. The defervescing DwoWS patients also had increased levels of liver enzymes AST, ALT and Gamma-GT. Raised AST levels during acute liver damage, has been associated with secreted IL-1ra which is an acute phase protein [49] produced by liver cells and also with IL-10, an anti-inflammatory cytokine which have previously correlated to necroinflammatory activity in liver damaged hepatitis C patients [50]. ALT, an enzyme present in hepatocytes was linked to IP-10 which is known to be induced in the liver. This cytokine plays a specific role in the intralobular accumulation of mononuclear cells and/or the death of hepatocytes in chronic hepatitis [51]. The gamma-GT levels, on the other hand were associated with IL-4, IL-12 and IL-9. Schistosomal patients with hepatic damage were found to have high levels of IL-4 [52] indicating an active T_h_2 immune response which could also be the situation in dengue patients. Elevated levels of IL-12 have been shown to be associated with liver damage in various studies conducted [53], [54]. At the convalescence phase, these without warning signs patients who had high levels of monocytes were associated with the increased levels of VEGF which possibly could be due to the involvement of VEGF in monocytes activation [55]. Surprisingly though, VEGF was inversely correlated to neutrophils. These patients also had high AST levels which was negatively associated with the decreasing levels of MCP-1 which have been implicated in the liver injury process [56].

Dengue patients with warning signs also exhibited thrombocytopenia, however, this clinical feature began earlier during the febrile phase and lasted till defervescence. A total of 6 different cytokines were thought to have possible association with platelet destruction, with 3 (IL-7, IL-12 and PDGF) occurring at both febrile and defervescence. IL-12 has been known to stimulate platelet-activating factor (PAF) [57] and the decrease of the cytokine probably disallowed normal platelet aggregation and degranulation. As mentioned earlier, the platelets are also known to release several growth factors, which probably explain the decreased levels of both VEGF and PDGF in the event of thrombocytopenia. Elevated levels of IL-7, conversely, have been previously shown to be involved in thrombocytosis [58]. During defervescence, the low levels of IL-5 probably deregulated functional PAF on eosinophils [59]. The presence of atypical lymphocytes in patients with warning signs from defervescence onwards was associated with G-CSF and IP-10.

The AST enzyme in dengue patients with warning signs was raised throughout the illness, and this enzyme was associated (i) at the febrile phase- ICAM-1, FGF-Basic, IL-13, IL-4, IL-12 and VEGF; (ii) defervescence: PDGF and (iii) convalescence- IFN-γ and IP-10. ICAM-1 has been suggested to play a role in inflammatory liver diseases [60] by recruiting leukocytes which can injure tissue by releasing various proteases and oxidants. The anti-inflammatory cytokines, IL-13 and IL-4 has been known to have hepatoprotective effects [61], [62]. IL-12 overexpression, as pointed out earlier, had been involved in liver damage, and many of its effects has been implied to be mediated by IFN-γ [53]. IFN-γ has also been suggested to be a negative regulator in liver cell proliferation and also to aggravate hepatitis viral-induced liver damage [63]. In an adult liver, endothelial cells provide nutritional and trophic support [64] and these cells are activated by VEGF and PDGF, whereby an association of decreased levels of VEGF was noticed with elevated levels of AST.

The main limitation in our study was the sample size number, and even though all patients were accounted and tested for cytokine levels at every phase of illness, some patients had undetected response towards certain cytokines. Furthermore, we did not include the severe dengue cases in our analyses as there were only four of them, hence losing out on valuable information as we could not establish cytokine trends/profile for severe dengue patients. Despite all these, our findings managed to re-establish the roles and dynamism of multiple cytokines at different phases of illness according to the new WHO dengue classification. The cytokine profiles from this study not only may have provided probable prognostics markers for but also shed new insights in dengue pathogenesis and this warrants further study. With the recent advancement of cytokine adjuvants and anti-cytokine therapies, our findings may serve towards better management in the field of dengue which is currently lacking a vaccine.
